# Skeletal muscle healing by M1-like macrophages produced by transient expression of exogenous GM-CSF

**DOI:** 10.1186/s13287-020-01992-1

**Published:** 2020-11-06

**Authors:** Leonardo Martins, Camila Congentino Gallo, Tâmisa Seeko Bandeira Honda, Patrícia Terra Alves, Roberta Sessa Stilhano, Daniela Santoro Rosa, Timothy Jon Koh, Sang Won Han

**Affiliations:** 1 Interdisciplinary Center for Gene Therapy, Escola Paulista de Medicina, Universidade Federal de São Paulo, São Paulo, Brazil; 2 Department of Physiological Sciences, Faculdade de Ciências Medicas da Santa Casa de São Paulo, São Paulo, Brazil; 3 Department of Microbiology, Immunology and Parasitology, Escola Paulista de Medicina, Universidade Federal de Sao Paulo, São Paulo, Brazil; 4 Department of Kinesiology and Nutrition, University of Illinois at Chicago, Chicago, USA; 5 Department of Biophysics, Escola Paulista de Medicina, Universidade Federal de São Paulo, Rua Mirassol 207, São Paulo, SP 04044-010 Brazil

**Keywords:** Fibrosis, Skeletal muscle, Injury, Myogenesis, GM-CSF, Macrophage, Arteriogenesis, Contusion

## Abstract

**Background:**

After traumatic skeletal muscle injury, muscle healing is often incomplete and produces extensive fibrosis. The sequence of M1 and M2 macrophage accumulation and the duration of each subtype in the injured area may help to direct the relative extent of fibrogenesis and myogenesis during healing. We hypothesized that increasing the number of M1 macrophages early after traumatic muscle injury would produce more cellular and molecular substrates for myogenesis and fewer substrates for fibrosis, leading to better muscle healing.

**Methods:**

To test this hypothesis, we transfected skeletal muscle with a plasmid vector to transiently express GM-CSF shortly after injury to drive the polarization of macrophages towards the M1 subset. C57BL/6 mouse tibialis anterior (TA) muscles were injured by contusion and electroporated with uP-mGM, which is a plasmid vector that transiently expresses GM-CSF. Myogenesis, angiogenesis, and fibrosis were evaluated by histology, immunohistochemistry, and RT-qPCR; subpopulations of macrophages by flow cytometry; and muscle functioning by the maximum running speed on the treadmill and the recovery of muscle mass.

**Results:**

Muscle injury increased the number of local M1-like macrophages and decreased the number of M2-like macrophages on day 4, and uP-mGM treatment enhanced this variation. uP-mGM treatment decreased TGF-*β*1 protein expression on day 4, and the Sirius Red-positive area decreased from 35.93 ± 15.45% (no treatment) to 2.9% ± 6.5% (*p* < 0.01) on day 30. uP-mGM electroporation also increased *Hgf*, *Hif1α*, and *Mtor* gene expression; arteriole density; and muscle fiber number during regeneration. The improvement in the quality of the muscle tissue after treatment with uP-mGM affected the increase in the TA muscle mass and the maximum running speed on a treadmill.

**Conclusion:**

Collectively, our data show that increasing the number of M1-like macrophages immediately after traumatic muscle injury promotes muscle recovery with less fibrosis, and this can be achieved by the transient expression of GM-CSF.

## Introduction

Muscle injuries often require medical care and are difficult to treat, resulting in absences from and disabilities at work and causing a significant public health burden [1]. In sports medicine, muscle injuries are the most frequent event among athletes in both recreational and competitive activities, reaching 55% of all cases [2]. Despite advances in diagnosis and therapy, treatment of muscle injury still primarily relies on RICE (rest, ice, compression, and elevation), immobilization, and physiotherapy [3, 4]. However, following severe muscle injury, patients suffer contracture, muscle atrophy, and reinjury because of incomplete muscle regeneration [2, 5]. Thus, advanced therapeutic methods are needed.

The healing process in the injured skeletal muscle consists of overlapping phases of degeneration, inflammation, regeneration, and fibrosis [2, 6]. Efficient regeneration of the injured muscle is thought to compete with fibrotic healing, and excessive fibrosis is thought to impede regeneration. This balance depends mainly on the cells and factors that are present at the degeneration and inflammation stages of healing. However, much remains to be learned about the regulation of this balance between regeneration and fibrosis.

Macrophages are the main players in the maintenance of tissue homeostasis and actively participate in tissue repair through both proinflammatory and anti-inflammatory activities [7, 8]. The apparent paradoxical activities of macrophages are due to the presence of heterogeneous subpopulations, each of which performs specific activities, and the plasticity of these cells in changing phenotypes in response to the local microenvironment and growth factor signaling [8, 9]. Inflammatory stimuli, such as IFN-γ and lipopolysaccharide, produce classically activated macrophages, also known as M1 macrophages, and anti-inflammatory stimuli, such as IL-4 or IL-13, produce alternatively activated macrophages, also known as M2 macrophages [8, 9].

As skeletal muscle is subjected to frequent injuries, several studies have shown the participation of macrophages in inflammatory, fibrotic, and regenerative processes [10, 11]. Soon after muscle injury, the number of M1 macrophages rapidly increases in the injured area, and approximately 2 days later, this population is replaced by M2 macrophages [9]. In vitro studies have shown that M1 macrophages reduce collagen production via fibroblasts and stimulate myoblast proliferation [10, 12, 13], while M2 macrophages increase collagen production and promote myoblast differentiation and fusion to form myofibers [10, 14]. These facts imply that the order of M1 and M2 macrophage production and the duration of the activity of each subtype in the injured area are directly related to the extent of fibrogenesis and myogenesis, and any significant changes in these processes can result in more fibrosis and less regenerated muscle or vice versa.

Based on these observations, we hypothesized that an additional increase in the number of proinflammatory macrophages for a few days at the beginning of muscle injury should produce more myoblasts and fewer myofibroblasts, providing more substrates for myogenesis and fewer substrates for fibrosis for the emerging proregenerative macrophages. To test this hypothesis, we transfected skeletal muscle with a plasmid vector to transiently express GM-CSF (granulocyte and monocyte colony stimulating factor) soon after injury because GM-CSF is known to polarize macrophages towards a proinflammatory phenotype [15, 16]. As a consequence, we expected to observe rapid muscle recovery with less fibrosis.

## Methods

### Research ethics committee approval

All the animal procedures were performed in full compliance with the institutional guidelines and were approved by the Institutional Animal Care and Use Committee (number: 8866221018).

### Murine GM-CSF expression vector

The murine GM-CSF (mGM-CSF) expressing plasmid vector was previously constructed in our laboratory and was named uP-mGM [17]. In this vector, mGM-CSF gene expression is promoted by the strong cytomegalovirus promoter and enhancer. For all experiments, the uP-mGM was amplified and purified using endotoxin-free plasmid purification systems (Qiagen Inc., Valencia, CA, USA).

### Skeletal muscle injury and in vivo gene therapy

Muscle contusion was performed based on the method described by Kasemkijwattana et al. [6]. C57/BL6 mice aging 10–12 weeks and weighting 25 g were anesthetized with an IP injection of ketamine (40 mg/kg of animal) and xylazine (10 mg/kg of animal). The mouse was positioned in ventral decubitus and the posterior limbs positioned extending the knee and plantar flexion. A stainless-steel ball weighting 16.2 g with 1.6 cm diameter was placed on the top of a PVC tube with 100 cm height to fall by gravity. At the bottom of the tube, a blunt-tipped nail (tip area is approximately equal to the TA muscle surface) was placed so that the impact was transferred to the entire TA muscle area. During first 4 days after contusion, fentanyl (0.05 mg/kg; IP) was administered to alleviate pain. Nonsteroidal anti-inflammatory drugs (NSAIDs) were not used here because these drugs affect muscle regeneration [18].

For in vivo transfection, electroporation was performed based on our previous experience that generates high transfection rate [19, 20]. Briefly, mice were anesthetized as described above and 100-μg plasmids in 100 μL PBS (137 mM NaCl, 10 mM Na_2_HPO_4_, 2 mM KH_2_PO_4_, pH 7.3) were injected intramuscularly using an insulin syringe. A pair of needle electrodes was introduced flanking the plasmid injected area, and 3 pulses of 80 V with 20 ms duration and 1 s intervals between pulses were applied (Eletroporator T820—BTX Genetronics, San Diego, USA). After electroporation, the mice were kept in the microisolators with ad libitum access to food and water.

### Histology

Mice were euthanized by cervical dislocation, and tibialis anterior (TA) muscles were collected for histology following previously established protocols [21]. Paraffin-embedded tissues were sectioned (4.5 μm) and stained with hematoxylin-eosin and Sirius Red. Silanized glass slides were used for immunohistochemistry (IHC) using the antibodies shown in Additional file 1. For histomorphometry, all images were captured inside injured areas and 20 randomly selected 200× or 400× high-powered fields from each sample. The percentages of cells expressing each marker of S1 were obtained after image acquisition with an optical microscope (Olympus BX60, Center Valley, PA, USA) and quantified using Image-Pro Plus® (*Media Cybernetics*, Rockville, MD, USA) according to a previously described protocol [21]. Pre-determined histomorphological criteria (see Additional file 2) were used for histomorphology.

### Real-time RT-PCR for quantitative gene expression analysis

Mice were euthanized by cervical dislocation to collect TA muscles. Total RNA from TA muscles was extracted using RNeasy® Microarray Tissue, and the cDNA was synthesized using RT^2^ First Strand Kit (Qiagen). For RT-qPCR, we used PCR array provided by the Qiagen (see Additional file 3). GAPDH gene was used as internal control. Each biological sample had a single replicate reaction, and two biological samples were used to all of them. Relative gene expression was calculated by 2^-ΔΔCT^. The changes in mRNA expression were expressed as fold changes relative to control.

### Flow cytometry

The TA muscles of the right and left limbs were collected on days 0, 4, 7, and 15 after injury, combined or not with electroporation with uP-mGM (100 μg/100 μL PBS), as described above. Muscles were incubated with 0.2% collagenase 1A solution (Sigma Aldrich) at 30 °C/45 min. Collagenase activity was blocked by the addition of 5 mL DMEM:FBS (1:1). The samples were filtered in cell strainer 70 and 40 μm.

Isolated cells were washed and resuspended with 0.5% PBS-BSA and preincubated with anti-CD16/32 antibody (eBioscience). Cells were incubated at 4 °C/30 min with the following antibodies: eFluor 450 anti-CD45 (clone 30-F11, eBioscience, catalog number 48-0451-82), FITC anti-F4/80 (clone BM8, eBioscience, catalog number 11-4801-82), APC-eFluor 780 anti-MHCII (clone M5/114.15.2, eBioscience, catalog number 47-5321-82), and PE anti-CD206 (clone C068C2, BioLegend, catalog number 141706). Dead cells were excluded by Fixable Viability Dye eFluor 506 (eBioscience) labeling and fixed in 1% buffered PFA for 15 min. Flow cytometry was performed using FACSCanto II (4.2.2) (BD Bioscience), and data were analyzed using FlowJo software (version 9.2). In this study, CD45+F4/80+MHCII+CD206− macrophages were considered as M1-like [22–24] and CD45+F4/80+MHCII−CD206+ as M2-like [23–25].

### Muscle function testing

Running capacity was tested in all groups. All animals underwent a 5-day motorized treadmill adaptation period (Exer 3/6 Treadmill, Columbus Instruments, Ohio, USA) prior to random separation between groups. Once a day, they ran for 15 min at a speed of 10 m/min. In addition to allowing the adaptation of the animals of the exercised groups to the training and handling conditions, this period also allowed to select the non-runner running animals. After this time, we performed the forced exercise capacity, which allowed to infer the physical performance of the animals by determining the maximum speed reached. The test was performed 1, 4, 7, 15, and 30 days after the last session of adaptation. Mice were then tested to exhaustion, defined as the point when mice would no longer run for more than 2 s at a time preferring to sit on the shock grid even when touched by a gloved hand. The steps of the test protocol were as follows: mice were started running at 5 m/min for the first 3 min, which increased to 10 m/min for 1 min, then increased by 1 m/min increments every 3 min thereafter until exhaustion [26, 27]. After this, all animals had a rest period of 24 h before any experimental procedure. After euthanasia of the animals, the tibialis anterior (TA) muscle was excised and its mass measured in an analytical balance.

### Statistical analysis

All the statistical analyses were performed in GraphPad Prism (Version 6.0 GraphPad Software Inc., San Diego, USA). The results are expressed as the mean ± SD. Differences were considered statistically significant at *p* < 0.05. The statistical methods used are detailed in the figure legends.

## Results

### Effects of injury and transfection on the expression of GM-CSF and its receptors

**Fig. 1:**
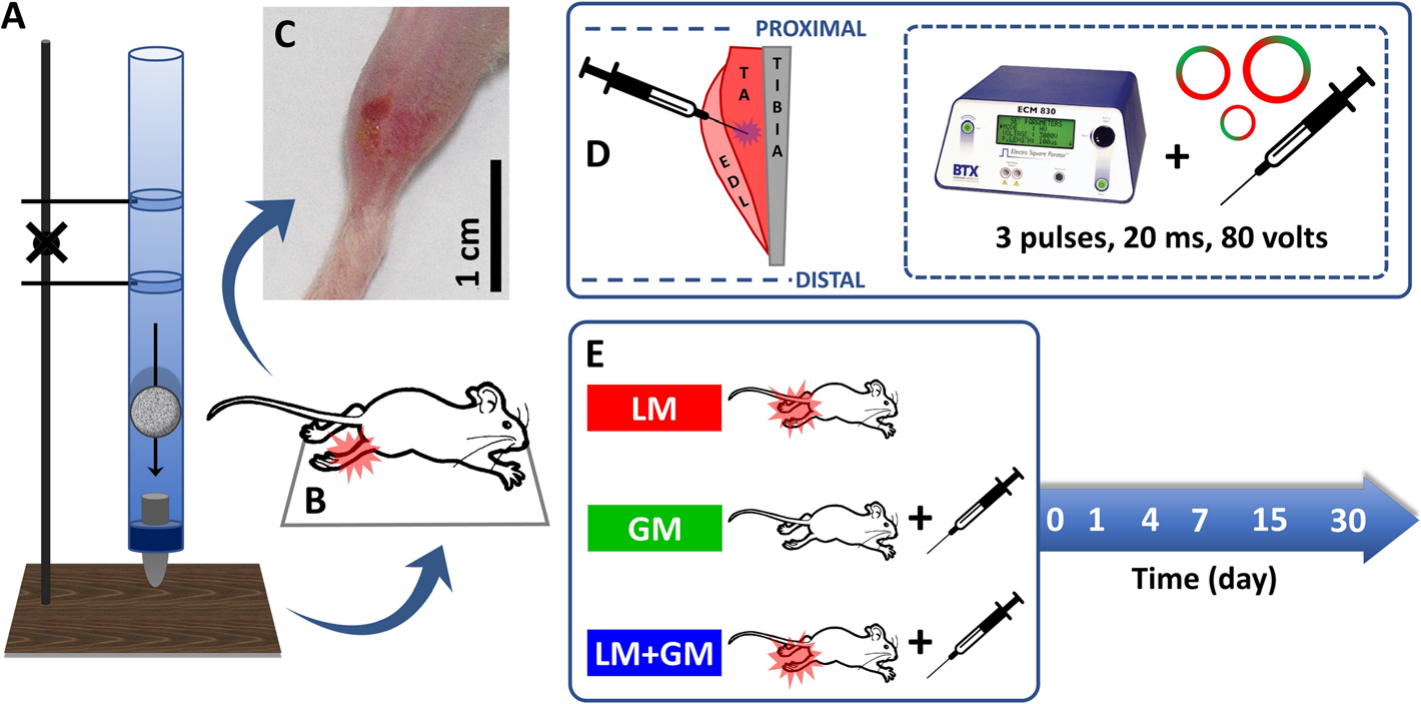
Illustration of skeletal muscle injury and gene therapy. (**a**) The height of the device was adjusted to 100 cm to transfer the 16.2-g projectile to the (**b**) tibialis anterior (TA) muscle of the animal placed in the supine position. (**c**) Representative image of an injured leg after 24 h and (**d**) representative scheme of the injection of the plasmid vectors into the central portion of the TA muscle; the overlapping structures, extensor digitorum longus (EDL), and tibial bone are also shown. (**e**) Experimental timeline and animal groups: GM, healthy mouse treated with GM-CSF; LM, injured mouse without treatment; LM+GM, injured mouse treated with GM-CSF

Mouse TA muscles were subjected to traumatic injury and transfected with uP-mGM vectors via electroporation, as shown in the schematic diagram in Fig. 1. The injured area was red and swollen soon after impact, but no sign of bone fracture was observed during the manual examination. The swelling lasted approximately 4 days, and the reddened area completely disappeared on the 7th day.

**Fig. 2:**
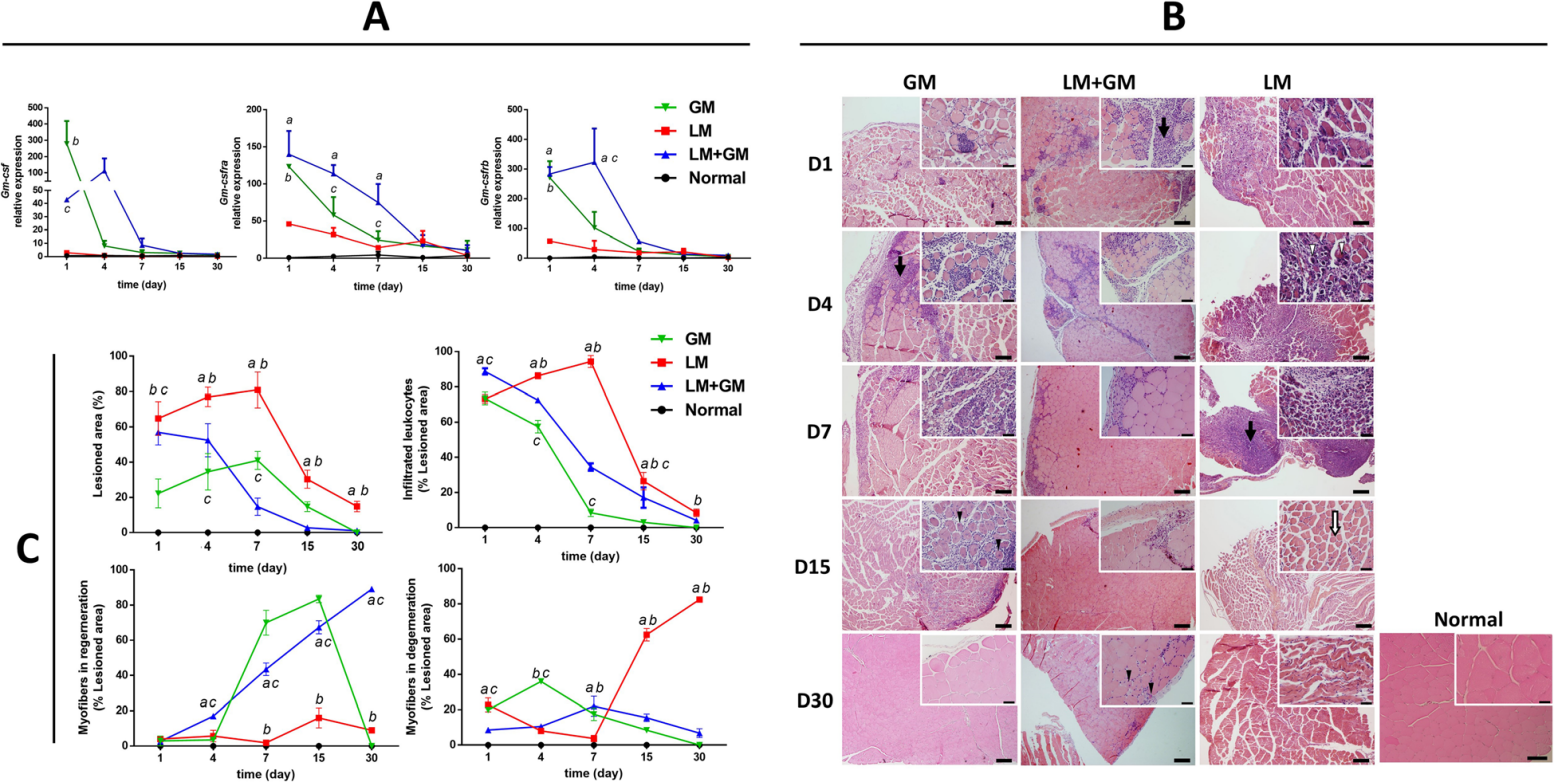
uP-mGM transfection promotes decreased inflammation. **a** Relative gene expression of GM-CSF and the GM-CSFR2 receptor assessed by quantitative RT-PCR. Two-way ANOVA and Tukey’s post hoc test. “a” *p* < 0.05 LM vs LM+GM; “b” *p* < 0.05 LM vs GM; “c” *p* < 0.05 LM+GM vs GM. **b** Micrographs of TA skeletal muscle tissue stained with HE. GM, healthy mouse treated with GM-CSF; LM, injured mouse without treatment; LM+GM, injured mouse treated with GM-CSF. Bar = 100 μm. Insets show × 400 magnified areas are shown in each figure. Bar = 50 μm. Leukocyte infiltrate, black arrow; atrophy, white arrow; cells with central nucleus, black arrowhead; cell necrosis, white arrowhead. **c** Histomorphometry was carried out using 20 images within the injured area per slide of each animal after HE staining. Data are presented in % (mean ± SD) of each parameter/total analyzed area. Two-way ANOVA and Tukey’s post hoc test. “a” *p* < 0.05 LM vs LM+GM; “b” *p* < 0.05 LM vs GM; “c” *p* < 0.05 LM+GM vs GM

The expression of *Gm-csf* and its receptors was analyzed by RT-qPCR. Healthy TA muscles expressed very low levels of *Gm-csf* and its receptors (*Gm-csfra* and *Gm-csfrb*). As expected, the expression of *Gm-csf* was dramatically increased 24 h after electroporation (GM) (279.41 ± 139.34-fold). Interestingly, the expression of *Gm-csfra* and *Gm-csfrb* was also increased (123.21 ± 1.28- and 270.77 ± 55.50-fold, respectively). The expression of these genes returned to basal levels after 1 week (Fig. 2A). When TA muscles were subjected to both injury and uP-mGM electroporation (LM+GM), the *Gm-csf* expression profile was similar to that of the GM group, but it lasted longer. The *Gm-csf* receptors in the LM+GM group were expressed at higher levels than those in the GM group and followed a similar decay. Injury itself also induced the expression of the *Gm-csf* receptors, but the levels were well below those induced by transfection with uP-mGM.

### Effect of uP-mGM transfection on muscle degeneration, inflammation, and regeneration

Muscle healing after injury was evaluated by quantifying the degenerated and regenerated muscle fibers, the injured muscle area, and the number of infiltrated leukocytes in HE-stained muscle sections, following previously defined criteria [21] (Fig. 2B, C and Additional file 2). Following injury alone (LM), progressive increases in the number of infiltrated leukocytes and the injured area were observed up to day 7, followed by rapid decreases (Fig. 2C). Electroporation of uP-mGM into non-injured muscle (GM) also resulted in some leukocyte infiltration and muscle damage but too much lesser extents than those observed in traumatic injury. Importantly, uP-mGM electroporation into traumatically injured muscle (LM+GM) led to a significant reduction in the injured area starting on day 4, and the injured area reached basal levels on day 15. uP-mGM electroporation also reduced the number of infiltrated leukocytes compared to traumatic injury alone.

Within the lesion area, the LM group showed a predominance of degenerated muscle fibers after day 7, while the LM+GM group showed very low levels of degenerated fibers and high levels of normal and regenerating muscle fibers. Within the smaller lesions of the muscles transfected with GM-CSF alone (GM), normal and regenerated fibers were abundant. Healthy muscles electroporated with uP vector did not significantly alter the quality and quantity of the muscle fiber (see Additional file 4). Thus, GM-CSF transfection appears to promote a regenerative healing response.

### Effect of uP-mGM transfection on muscle fibrosis analysis

**Fig. 3:**
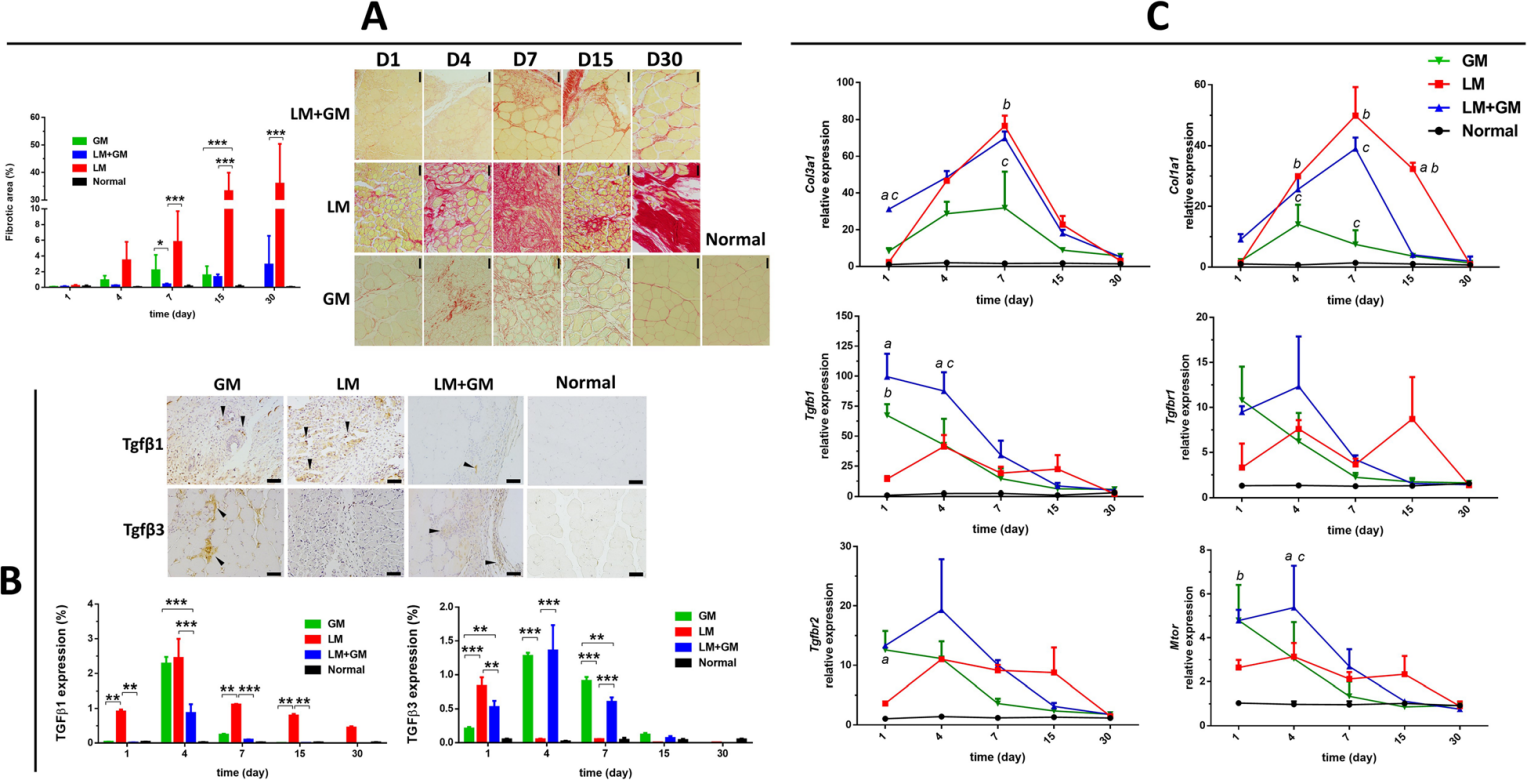
uP-mGM transfection promotes decreased fibrosis and alters the fibrosis-related gene expression profile. **a** Micrographs of TA skeletal muscle tissue stained with Picrosirius Red and histomorphometry carried out using 20 images within the injured area per slide of each animal after staining. Data are presented in % (mean ± SD) of each parameter/total analyzed area. Bar = 50 μm. Two-way ANOVA and Tukey’s post hoc test. *p < 0.5, ***p < 0.001. **b** Expression of TGF-β1 and TGF-β3 was assessed by IHC, and histomorphometry was carried out using 20 images per slide of each animal after staining. Bar = 50 μm. Two-way ANOVA and Tukey’s post hoc test. **p < 0.1, ***p < 0.001. **c** Fibrogenic gene expression analyzed by RT-qPCR. Two-way ANOVA and Tukey’s post hoc test. “a” *p* < 0.05 LM vs LM+GM; “b” *p* < 0.05 LM vs GM; “c” *p* < 0.05 LM+GM vs GM

To analyze fibrosis, we first performed Sirius Red staining to evaluate the total collagen content. In healthy skeletal muscle, the Sirius Red-stained area was minimal, but after injury, the stained area progressively increased, reaching 35.93 ± 15.45% of the muscle area on day 30 (Fig. 3A). Thus, the contusion injury model results in significant skeletal muscle fibrosis. Importantly, GM-CSF treatment (LM+GM) significantly inhibited fibrosis, as the Sirius Red-stained area was maintained close to that observed at baseline. GM-CSF overexpression in healthy muscle did not significantly affect fibrosis.

To better understand the mechanisms involved in fibrosis after traumatic injury, muscle sections were stained with anti-TGF-*β*1 and TGF-*β*3 antibodies (Fig. 3B). In the LM mice, the TGF-*β*1-stained area increased quickly after injury and then decreased over time. GM-CSF treatment drastically reduced the TGF-*β*1-stained area. However, the TGF-*β*3-stained area showed an inverse response to GM-CSF compared to the TGF-*β*1-stained area (Fig. 3B). LM muscles exhibited a peak expression on day 1, which subsequently disappeared, but the GM-CSF-treated (LM+GM) muscles showed significantly enhanced TGF-*β*3 levels on days 4 and 7 postinjury. The expression of GM-CSF in healthy muscle (the GM group) generated TGF-*β*1 and 3 profiles that were similar to those generated by the expression of GM-CSF in injured muscle (the LM+GM group). In summary, the expression profile of TGF-*β*1 is compatible with the promotion of fibrosis observed by Sirius Red staining, whereas the expression profile of TGF-β3 is associated with antifibrosis.

The expression of fibrosis-related genes was also assessed by RT-qPCR. The expression of *Tgfb1* rapidly increased after muscle injury (LM) and returned to baseline by day 30. Transfection of uP-mGM into healthy muscle also caused a rapid increase in the expression of *Tgfb1*. Transfection into the injured muscle (LM+GM) resulted in an expression profile over time that was similar to that induced by transfection into healthy muscle, but the expression levels tended to be amplified. On the other hand, the expression of the TGF-β1receptors *Tgfbr1* and *Tgfbr2* was high in all the groups only during the first 7 days, and only the LM group maintained high gene expression levels thereafter, while the other groups maintained baseline gene expression levels (Fig. 3C). Therefore, the expression profiles of TGF- β1 mRNA and protein were not similar.

*Col1a1*, which is a marker of fibrosis, was overexpressed in traumatically injured muscle, reaching a peak at day 7 and slowly returning to baseline at day 30. The expression of *Col1a1* was significantly reduced in the LM+GM group on days 7 and 15 postinjury (Fig. 3C). In contrast, the *Col3a1* gene expression profile was similar between the LM and LM+GM groups, and the expression in both groups peaked on day 7 and returned to baseline on day 30.

mTOR is a key regulator in the maintenance of skeletal muscle and regeneration after injury. Muscle injury elevated *Mtor* expression, but transfection with uP-mGM promoted much higher *Mtor* expression, which returned to baseline levels after 1 week.

### Effect of uP-mGM transfection on angiogenesis

**Fig. 4:**
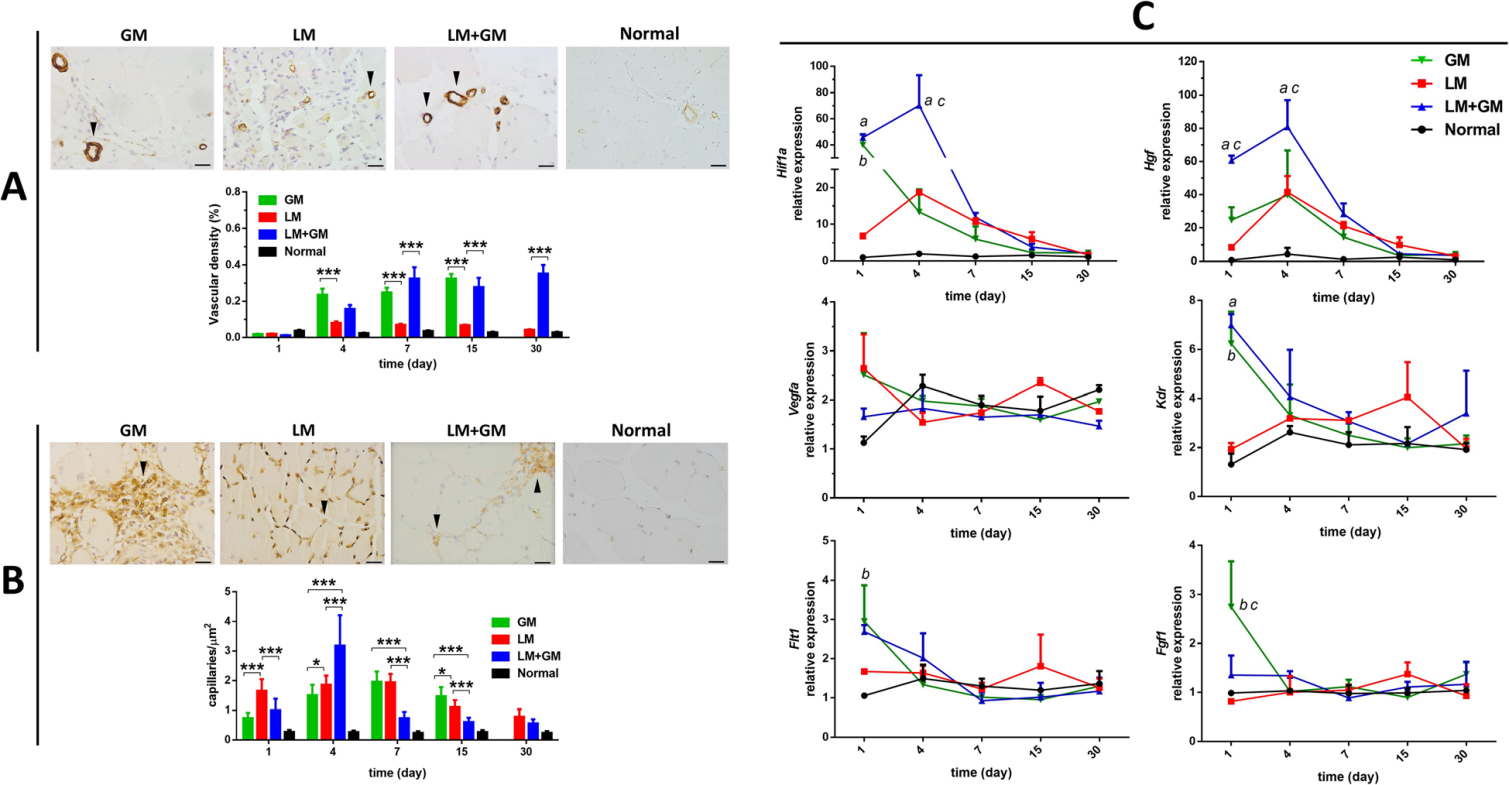
uP-mGM transfection promotes increased GM-CSF receptor expression and vessel density. **a** Micrographs of TA skeletal muscle tissue stained with an α-smooth muscle actin (α-SMA) antibody. Histomorphometry was carried out using 20 frames within the injured area per slide of each animal after staining. Bar = 20 μm. Two-way ANOVA and Tukey’s post hoc test. ***p < 0.001. Arrow heads indicate large vessels stained with α-SMA. **b** Micrographs of the same sample stained with Isolectin IB4 Biotin. Histomorphometry was carried out using 20 frames within the injured area per slide of each animal after staining. Bar = 20 μm. Two-way ANOVA and Tukey’s post hoc test. *p < 0.5, ***p < 0.001. Arrow heads indicate capillaries. **c** Angiogenic gene expression analyzed by RT-qPCR. Two-way ANOVA and Tukey’s post hoc test. “a” *p* < 0.05 LM vs LM+GM; “b” *p* < 0.05 LM vs GM; “c” *p* < 0.05 LM+GM vs GM

Angiogenesis was evaluated in muscle sections by identifying vessel-like structures using an anti-α-SMA antibody (arterioles) and Isolectin IB4 staining (capillaries). Traumatic injury alone (LM) resulted in very few α-SMA-positive vessels at all time points, whereas uP-mGM transfection resulted in increased a-SMA-positive vessels beginning on day 4, which persisted until day 30 in both the LM and LM+GM groups (Fig. 4A).

Measurements of capillary density showed similar trends among the LM, LM+GM, and GM groups, reaching a peak on day 4 and decreasing to baseline on day 30. Among all the time points investigated, uP-mGM transfection only increased the capillary density on day 4 postinjury. Therefore, uP-mGM transfection appears to have primarily promoted arteriogenesis and to have a smaller effect on angiogenesis.

The expression of angiogenic genes was assessed by RT-qPCR using muscle samples from each time point. Among the 6 genes (*Hif1a*, *Hgf*, *Kdr*, *Flt1*, *Vegfa*, and *Fgf1*) assessed, traumatic injury (in the LM and LM+GM groups) induced a significant increase in the expression of *Hif1a* and *Hgf*, which reached a maximum value on day 4. Importantly, transfection with uP-mGM produced a further significant increase in these genes.

In addition, transfection with uP-mGM increased the expression of the *Vegfa* receptor *Kdr* on day 1 after electroporation in both the healthy and LM groups, but this expression returned to baseline on day 4. Therefore, the increase in α-SMA-positive vessel density could be related to the increase in HIF*α*, which is the main regulator of angiogenesis [28], and HGF, which promotes arteriogenesis and angiogenesis [29].

### Effect of uP-mGM transfection on macrophage subpopulations

**Fig. 5:**
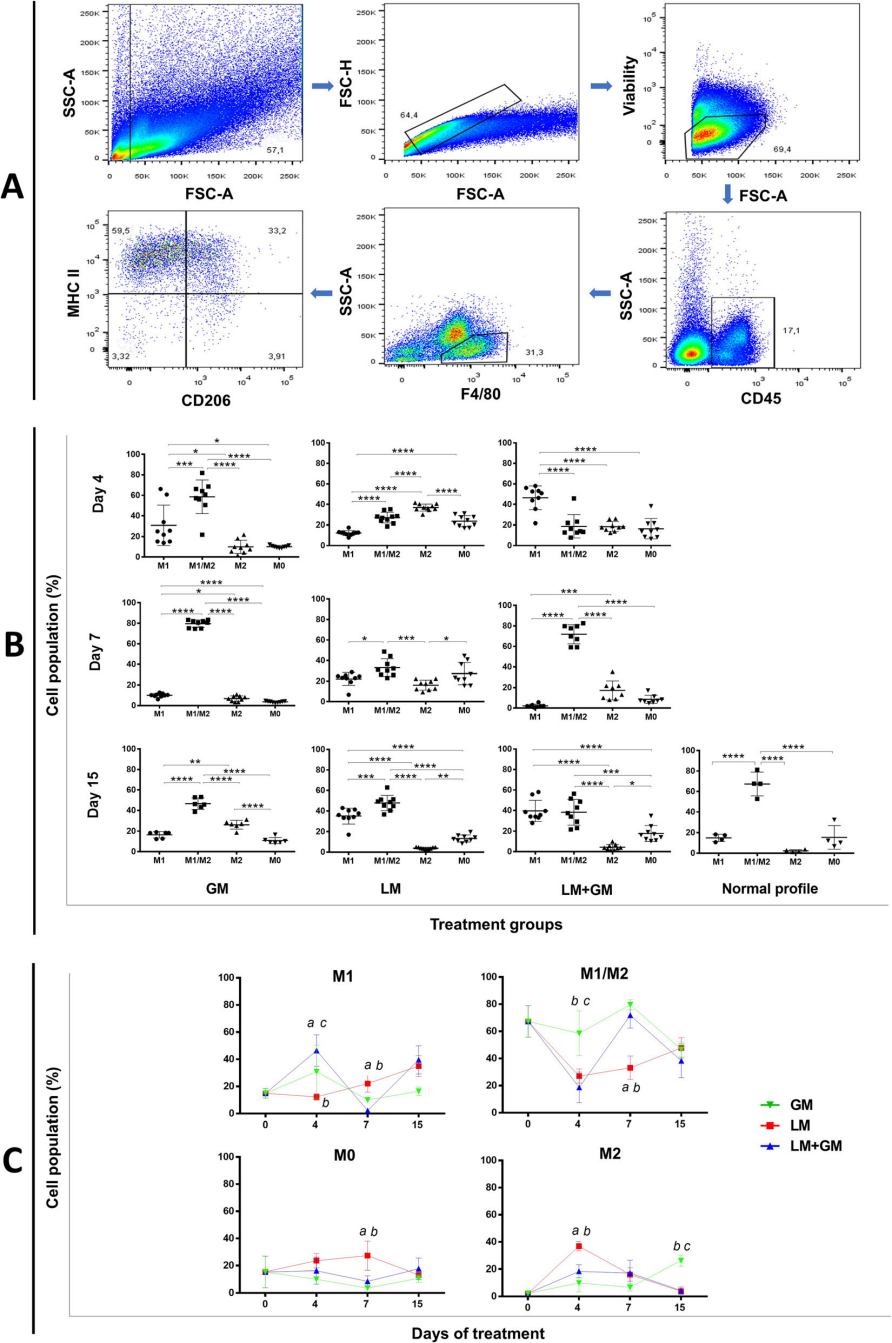
uP-mGM transfection leads to increased numbers of the M2 macrophage subtype. **a** Flow cytometry analysis of the macrophage subpopulations. Gating strategy used to identify M0 (CD45^+^ F4/80^+^ CD206^−^ MHCII^−^), M1 (CD45^+^ F4/80^+^ CD206^−^ MHCII^+^), M2 (CD45^+^ F4/80^+^ CD206^+^ MHCII^−^), and M1/M2 (CD45^+^ F4/80^+^ CD206^+^MHCII^+^) cells. **b** Macrophage subpopulations in the LM, GM, and LM+GM groups over 15 days. The normal group represents mice that did not undergo injury and treatment. **c** Variation in the numbers of macrophages per subpopulation over time. Time zero is represented by macrophages from the normal group, as mentioned above (**b**). Two-way ANOVA and Tukey’s post hoc test. “a” *p* < 0.05 LM vs LM+GM; “b” *p* < 0.05 LM vs GM; “c” *p* < 0.05 LM+GM vs GM

The macrophage subpopulations in the muscles were assessed by flow cytometry. First, we gated out debris in the SSC (side scatter) vs FSC (forward scatter) plots; then gated on singlets, live cells, and CD45+ and F4/80+ cells; and finally identified subpopulations using MHCII and CD206 as markers for M1-like and M2-like macrophages, respectively (Fig. 5A). A typical dot plot showed 4 distinct populations based on the expression of MHCII and CD206: MHCII^+^CD206^−^ (M1-like), MHCII^−^CD206^+^ (M2-like), MHCII^+^CD206^+^ (hybrid), and MHC^−^CD206^−^ (non-expressor). In non-injured muscle, most macrophages were MHCII^+^CD206^+^, with lower frequencies of MHCII^+^CD206^−^ and MHCII^−^CD206^−^ cells and a barely detectable number of MHCII^−^CD206^+^ macrophages (Fig. 5B, C). Muscle injury caused a rapid increase in the frequency of MHCII^−^CD206^+^ macrophages that peaked on day 4 and slowly decreased over time, reaching baseline on day 15. MHCII^+^CD206^−^ macrophages (Fig. 5B) behaved differently, slowly increasing after injury throughout the time points examined (Fig. 5C). In contrast, the MHCII^+^CD206^+^ macrophage population rapidly decreased on day 4 and recovered over time. Thus, MHCII^+^CD206^+^ macrophages may serve as a buffer, providing MHCII^+^CD206^−^ and MHCII^−^CD206^+^ cells, as these are required during the repair process. This speculation should be tested in future studies.

As expected, transfection with uP-mGM caused a rapid increase in the number of MHCII^+^CD206^−^ macrophages, which peaked on day 4 postinjury. GM-CSF overexpression in injured muscle amplified the increased numbers of this macrophage subset (day 4), which then decreased to baseline on day 7 and increased again on day 15 to a level similar to that observed in the LM group. Thus, it appears that the first increase in the numbers of MHCII^+^CD206^−^ macrophages is due to GM-CSF overexpression, and the second increase is due to injury.

### Muscle mass and functional recovery

As muscle injury affects muscle mass and function, a treadmill running performance test was used to assess muscle function, after which the TA muscles were harvested and weighed.

**Fig. 6:**
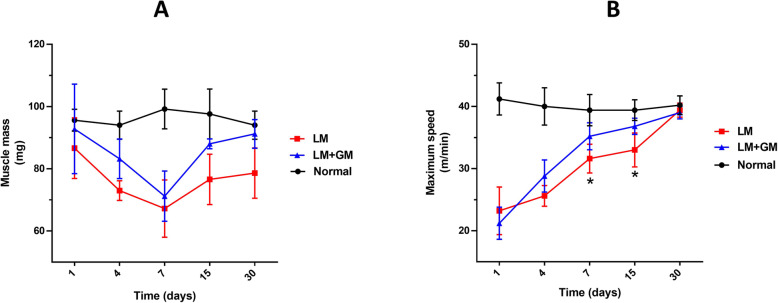
uP-mGM transfection promotes recovery of muscle functional. **a** TA muscle mass was determined 30 days after transfection. **b** Performance of incremental maximal exercise was assessed in mice forced to run to maximum exhaustion using a standardized protocol of incremental speeds and 0° inclination of the treadmill. Two-way ANOVA and Tukey’s post hoc test. *p < 0.05 LM vs LM+GM. Data presented as mean ± SD

Traumatic injury resulted in a progressive loss of muscle mass soon after injury, remarkably peaked on day 7, followed by gradual recovery (Fig. 6). Importantly, muscle mass did not recover to baseline levels by day 30 postinjury. GM-CSF-treated mice showed a similar loss in mass but a more complete recovery, reaching baseline values by day 30.

With regard to muscle function, the maximum treadmill running speed dramatically decreased after injury and returned to baseline by day 30 postinjury (Fig. 6).

The GM-CSF-treated group followed a profile similar to that of the LM group, but the recovery speed was faster. However, the variations in muscle mass and speed recovery did not exhibit statistical significance within groups at different adjacent time points or between the LM and LM+GM groups at each time point.

## Discussion

Full restoration of skeletal muscle structure and function after traumatic injury requires several different cell types and numerous molecules working together to efficiently control the damaged tissue through each phase of healing. Macrophages are known to play essential roles in each phase of efficient muscle regeneration, but the regulation of macrophage subsets may be dysregulated in traumatic injury, resulting in extensive fibrosis [10, 11].

GM-CSF polarizes macrophages towards a proinflammatory phenotype (see Additional file 5) [15, 16], and in our study, this phenomenon can be observed by the increase in M1-like macrophages and decrease in M2-like macrophages on day 4 (Fig. 5B, C). The variations in this cell population over 15 days were very similar in the GM and LM+GM groups but were enhanced in the LM+GM group. These results show that the balance of the M1-like and M2-like populations can be disrupted by GM-CSF, favoring the formation of more M1-like cells. It is also important to note that this disruption lasted only 1 week and peaked at day 4 after electroporation, which is in accordance with the GM-CSF expression profile (Fig. 2A). The non-integrative plasmid vector-mediated gene expression in skeletal muscle usually lasts approximately 1 week, and after this time, a very low level of expression is maintained [30]. Therefore, the temporal increase in the number of M1-like macrophages as a consequence of electroporation at the beginning of muscle injury achieves our initial goals, namely, more myogenic activity and less fibrogenic activity.

Here, we used the terms M1-like and M2-like, which indicate proinflammatory and anti-inflammatory macrophages, respectively, although we acknowledge that these subtypes are only points on a continuum of macrophage phenotypes. Most cell markers have been identified using cell cultures, and cell media are well defined; however, in vivo macrophage phenotypes are likely more complex because they are exposed to a complex and rapidly changing microenvironment following injury. For example, Novak et al. [11] reported that in traumatically injured muscle, macrophages do not exhibit the canonical M1 or M2 phenotypes, and M1- and M2a-associated genes are coincidentally upregulated soon after injury, leading to difficulty in classifying macrophages and variation in the results from different authors.

We chose GM-CSF to prove our hypothesis because in addition to stimulating the proliferation and differentiation of monocytes/macrophages and polarizing macrophages towards a proinflammatory phenotype [15, 16], GM-CSF also mobilizes endothelial progenitor cells for vasculogenesis [31] and stimulates the proliferation of neutrophils that express various angiogenic growth factors, such as HGF [32]. The increase in HGF expression (Fig. 4C) and vessel density (Fig. 4A) shows the roles of GM-CSF activities in the formation and remodeling of vessels to become larger and more functional, as has also been seen by others [33, 34]. HGF also triggers activation of satellite cells [35]; consequently, it participates in myogenesis. As GM-CSF regulates the development of dendritic cells, this factor has shown promise for cancer therapies [36, 37]. Thus, people who are concerned about the use of growth factor genes for angiogenic gene therapy, because these factors can stimulate tumorigenesis, are much less concerned about GM-CSF-based therapy. Therefore, a collection of information from different sources shows that GM-CSF can be a crucial factor to resolve injured skeletal muscle if appropriately administered.

The biological effects of GM-CSF can occur only if there is a sufficient concentration of GM-CSF and its receptor on the cell membrane. The GM-CSF receptor is composed of two subunits: subunit *α*, which binds with high affinity to GM-CSF, and subunit *β* c, which is responsible for signal transduction [38, 39]. This receptor is mostly expressed in myeloid cells, such as neutrophils, monocytes, and macrophages [38], and in dendritic cells [40]. When GM-CSF binds to its receptor, this complex is internalized to be recycled and partially degraded, and this process seems to stimulate synthesis of its receptors [41]. This observation can explain, at least in part, the temporal increase in the mRNA concentration of the GM-CSF receptor subunits in the muscle after electroporation (Fig. 2A).

It was shown that GM-CSF concentrations lower than 10 pM promote hematopoietic cell survival (extended lifetime) by the phosphorylation of the subunit βcSe^585^. However, if this concentration is increased above 10 pM, then this Ser is dephosphorylated, and βcTyr^577^ is phosphorylated, which increases cell survival and proliferation [42]. In our previous study, we observed 8.6 ± 3.8 ng of GM-CSF in a thigh muscle weighing 160 ± 23 mg after electroporation with uP-mGM [17]. Considering the physiological conditions and the molecular properties of GM-CSF, we observed a concentration of approximately 2.6 μM (see Additional file 6). This concentration is well above 10 pM; therefore, electroporation with uP-mGM must produce enough GM-CSF to stimulate the proliferation, survival, and functional activities of hematopoietic cells. A direct proof of this assumption is the increase in the number of macrophages on day 4 after electroporation into the healthy (GM) and injured-muscle (LM+GM) mice (Fig. 5B, C).

Resident macrophages are rare in healthy muscles and are located mainly in the epimysium and perimysium [43–46]. These resident macrophages likely have essential roles in the recruitment of circulating monocytes because the ablation of resident macrophages reduces monocyte recruitment to toxin-injured muscle [43]. As GM-CSF stimulates the proliferation of macrophages irrespective of origin, any increase in the number of local macrophages induced by GM-CSF overexpression likely includes a combination of resident and monocyte-derived macrophages. As our goal was to evaluate the effect of GM-CSF overexpression on macrophages present in the injured muscle regardless of origin, we used F4/80 as an initial marker and then used CD206 and MHCII as markers of M2-like and M1-like macrophages, respectively [24, 47, 48].

One of the main consequences of tissue injury is fibrosis, which is a common biological response to the rapid repair of damaged tissue. However, fibrosis affects tissue function, and in some cases, it can severely impair function and even lead to death, such as in the heart and kidney [49, 50]. As shown in Fig. 3, traumatically injured muscle developed exuberant fibrosis, which was almost completely reduced by GM-CSF overexpression. TGF-*β*1 is a main player in fibrosis [51–53]. TGF-*β*1 also participates in myoblast proliferation [54], but it also delays myoblast differentiation and fusion to make new myofibers [55]. Therefore, it seems that higher expression of TGF-*β*1 at the beginning of muscle injury and lower expression later promote more myogenesis and less fibrogenesis; this expression profile can be observed after GM-CSF treatment (Fig. 3C). The increase in TGF-*β*1 expression observed soon after uP-mGM transfection is probably due to stimulation by GM-CSF and autoregulation of TGF-*β*1 [56, 57]. The correlation between these two factors can be observed by the similarity of their RNA gene expression patterns (Figs. 2A and 3C). However, the TGF-*β*1 protein expression pattern observed by IHC is significantly different from the RNA gene expression pattern. Most likely, the antibody we used for IHC does not recognize one of the TGF-*β*1 forms (active and latent complex) well [58]. In addition, other factors, such as the different stabilities of the mRNA and protein of the same factor and the rate of translation, can cause variation in concentrations [59, 60]. Importantly, both data support the correlation between GM-CSF and TGF-*β*1 and fibrosis. The reduction in fibrosis can also be due to the elevation in expression of TGF-*β*3 (Fig. 3B), which belongs to the same TGF-*β* family but can reduce fibrogenesis [61]. The antifibrotic activity of TGF-*β*3 has also been used to treat skin scarring [62, 63].

Muscle mass homeostasis depends on metabolic rates, and several studies have shown that mTOR positively regulates muscle protein synthesis and, consequently, muscle hypertrophy [64]. In humans, mTORC1 activation induces myofibrillar muscle protein synthesis and muscle hypertrophy after physical exercise or ingestion of essential amino acids, such as leucine [65]. The involvement of mTOR was also shown in the regenerative process of severely injured muscle [66], which can also be observed in our injury model (Fig. 2C). Overexpression of GM-CSF enhanced the increase in mTOR expression, especially during the first week after electroporation, which should have significantly contributed to the increase in regenerating muscle fibers and the decrease in degenerating muscle fibers (Fig. 2C). The consequence of the increased mTOR expression and accelerated muscle recovery over time after GM-CSF administration was also reflected in the tendency to increase muscle mass (Fig. 6a) and the faster recovery of muscle function (Fig. 6b).

## Conclusion

Our data show that the temporal increase in the number of M1-like macrophages soon after traumatic muscle injury is important for the recovery of skeletal muscle with less fibrosis, and this can be achieved by the transient expression of GM-CSF.

## Supplementary Information

**Additional file 1.**

**Additional file 2.**

**Additional file 3.**

**Additional file 4.**

**Additional file 5.**

**Additional file 6.**

## Data Availability

All data generated and/or analyzed during this study are included in this published article.

## Abbreviations

GM-CSF: Granulocyte macrophage-colony stimulating factor
TA: Tibialis anterior
HE: Hematoxylin-eosin staining
IHC: Immunohistochemistry
PBS: Phosphate-buffered saline
RT-PCR: Reverse transcriptase-polymerase chain reaction
TGF-β1: *Transforming growth factor beta* 1
TGF-β3: *Transforming growth factor beta* 3
α-SMA: α-smooth muscle actin
uP-mGM: uP vector expressing murine GM-CSF
