# Supplementary material for: Skeletal muscle healing by M1-like macrophages produced by transient expression of exogenous GM-CSF
Source: Stem Cell Res Ther. 2020 Nov 6;11:473. doi: 10.1186/s13287-020-01992-1 (PMC7648431; doi:10.1186/s13287-020-01992-1)
Supplement: Supplementary file 1 — Additional file 1. [file 13287_2020_1992_MOESM1_ESM.docx]

**ADDITIONAL FILE 1**

**Antibodies used in IHC experiments**

| **Antibody** | **Dilution** | **Company** | **Reference** |
| --- | --- | --- | --- |
| anti-TGFβ1 (D-12) (sc-31608) | 1:100 | Santa Cruz Biotechnology, Inc., Dallas, TX, EUA | [1,2] |
|  |  |  |  |
| anti-TGFβ3 (V) (sc-82) | 1:100 | Santa Cruz Biotechnology, Inc., Dallas, TX, EUA | [3] |
|  |  |  |  |
| Isolectin B4 (B-1205)* | 1:50 | Vector Labs. Inc, Burlingame, CA, EUA | [4] |
|  |  |  |  |
| anti-α-SMA (1A4) | 1:100 | Dako, Carpinteria, CA, EUA | [3] |

*****Isolectin IB4 is a glycoprotein isolated from the seeds of the tropical African legume *Griffonia simplicifolia*.
