# Supplementary material for: Skeletal muscle healing by M1-like macrophages produced by transient expression of exogenous GM-CSF
Source: Stem Cell Res Ther. 2020 Nov 6;11:473. doi: 10.1186/s13287-020-01992-1 (PMC7648431; doi:10.1186/s13287-020-01992-1)
Supplement: Supplementary file 2 — Additional file 2. [file 13287_2020_1992_MOESM2_ESM.docx]

**ADDITIONAL FILE 2**

**Criteria used for histopathological analysis**

| Classification | Histological characterization after staining with HE and meaning | References |
| --- | --- | --- |
| Degeneration | Cellular swelling, hypereosinophilia, vacuolization, fibre fragmentation and rupture. | [5,6] |
| Necrosis | Presence of infiltrating inflammatory cells (myophagocytosis), fragmented sarcoplasm, dark coloured (hyper-laid fibres) and pale coloured (necrotic fibres) fibres. | [5,6] |
| Muscle atrophy | Reduction of myofiber diameter and hypereosinophilic sarcoplasm. | [5,6] |
| Inflammation | Presence of Polymorphonuclear neutrophilic (PMN), leukocyte and/or infiltrated leucocytes with varying morphological patterns | [6,7] |
| Regeneration | Fibres with centralized nucleus myocytes; Basophilic staining (active regeneration). | [5,6] |
| Fibrosis | Increased number of reactive fibroblasts with prominent vesiculated nuclei; Increased amount of pale eosinophilic fibrillary material (collagen deposition) separating and / or surrounds adjacent myofibers. | [8] |
