# Supplementary material for: Skeletal muscle healing by M1-like macrophages produced by transient expression of exogenous GM-CSF
Source: Stem Cell Res Ther. 2020 Nov 6;11:473. doi: 10.1186/s13287-020-01992-1 (PMC7648431; doi:10.1186/s13287-020-01992-1)
Supplement: Supplementary file 3 — Additional file 3. [file 13287_2020_1992_MOESM3_ESM.docx]

**ADDITIONAL FILE S3**

**Genes analysed by RT-qPCR experiment**

| **Gene name** | **Symbol** | **NCBI database reference** |
| --- | --- | --- |
| collagen, type I, alpha 1 | *Col1a1* | NM_007742.3 |
| collagen, type III, alpha 1 | *Col3a1* | NM_009930.2 |
| transforming growth factor, beta 1 | *Tgfb1* | NM_011577.1 |
| transforming growth factor, beta receptor I | *Tgfbr1* | NM_009370.2 |
| transforming growth factor, beta receptor II | *Tgfbr2* | NM_009371.3 |
| vascular endothelial growth factor A | *Vegfa* | NM 001025250.3 |
| FMS-like tyrosine kinase 1 | *Flt1 (VEGFR1)* | NM_010228.3 |
| kinase insert domain protein receptor | *Kdr*  *(VEGFR2)* | NM_010612.2 |
| fibroblast growth factor 1 | *Fgf1* | NM_010197.3 |
| hepatocyte growth factor | *Hgf* | NM_010427.4 |
| hypoxia inducible factor 1, alpha subunit | *Hif1a* | NM_010431.2 |
| colony stimulating factor 2 (granulocyte-macrophage) | *Gm-csf (Csf2)* | NM_009969.4 |
| colony stimulating factor 2 receptor, alpha, low-affinity (granulocyte-macrophage) | *Gm-csfra* | NM_009970.2 |
| colony stimulating factor 2 receptor, beta, low-affinity (granulocyte-macrophage) | *Gm-csfrb* | NM_007780.4 |
| Paired box 3 | *Pax3* | NM_008781.4 |
| Paired box 7 | *Pax7* | NM_011039.2 |
| myostatin | *Mstn* | NM_010834.2 |
| myogenic differentiation 1 | *Myod1* | NM_010866.2 |
| follistatin | *Fst* | NM_008046.2 |
| mechanistic target of rapamycin kinase | *Mtor* | NM_020009.2 |
| glyceraldehyde-3-phosphate dehydrogenase | *Gapdh* | NM_008084.2 |
