# Supplementary material for: Skeletal muscle healing by M1-like macrophages produced by transient expression of exogenous GM-CSF
Source: Stem Cell Res Ther. 2020 Nov 6;11:473. doi: 10.1186/s13287-020-01992-1 (PMC7648431; doi:10.1186/s13287-020-01992-1)
Supplement: Supplementary file 4 — Additional file 4. [file 13287_2020_1992_MOESM4_ESM.docx]

Micrographs of the skeletal muscle transfected with the empty vector uP in days (D) 1, 4, 7, 15 and 30, and stained with hematoxylin and eosin.

EP: electroporation. Bar = 50 μm. Inset figure bar = 25 μm.
