# Supplementary material for: Skeletal muscle healing by M1-like macrophages produced by transient expression of exogenous GM-CSF
Source: Stem Cell Res Ther. 2020 Nov 6;11:473. doi: 10.1186/s13287-020-01992-1 (PMC7648431; doi:10.1186/s13287-020-01992-1)

**Polarization of RAW 264.7 macrophages to M1-like macrophages by uP-mGM transfection**

RAW 264.7 macrophages were cultured in DMEM (Gibco, #12100-046) supplemented with 5% fetal bovine serum (Gibco, #12657-029) and 1% penicillin and streptomycin (Gibco, #15140-120) at 37 °C in a CO_2_ incubator with 5% CO_2_. Initially, 3 x 10^5^ cells were seeded per well in a 24-well plate containing a glass coverslip per well. After 16 h, cells were polarized M1 or M2 macrophages, or transfected with uP or uP-mGM vectors. These cells were assessed for iNOS expression, M1-macrophage marker, by immunocytochemistry and quantification of GM-CSF protein in the cell culture supernatant by ELISA.

Macrophage polarization was performed according to Weinstock, L et al. (2020). Briefly, the medium was replaced with a fresh medium containing 1μg/mL of lipopolysaccharide (LPS; Sigma-Aldrich; L2880) and 100 ng/mL IFN-γ (R&D Systems; 485-MI), or 50 ng/mL IL-4 (PeproTech; 214-14) and incubated for 48 h for polarization to M1 or M2 macrophages, respectively. For transfection, Lipofectamine ®2000 (Invitrogen, #11668-019) was used following the transfection protocol provided by the manufacturer, and cells were evaluated after 72 h by immunochemistry or ELISA, as shown below.

Cells were washed 3 times with PBS, fixed with 4% paraformaldehyde (PFA) in PBS for 15 min, permeabilized with 0.1% Triton X-100 for 5 min and blocked with 1% PBS/BSA for 1h. Cells were stained with anti-iNOS antibody (Invitrogen, #PA1-036, 1:100) for 3 h, followed with the anti-rabbit antibody conjugated to Alexa 488 (Invitrogen, #A11008, 1:200) for 1 h, and stained with DAPI (4′,6-diamidino-2-phenylindole, Thermo, #62247, 5μg.mL^−1^,) for 5 min. Samples were washed 3 times with PBS after each step. The analysis and acquisition of images were performed using the fluorescence microscope Axio Observer Z1 (ZEISS, Oberkochen, Germany).

Mouse GM-CSF produced by these cells were collected from supernatants and assessed by ELISA using the Mouse GM-CSF DuoSet ELISA kit (R&D system, #DY415), following the manufacturer’s protocol.

M0, M1, M2 and uP transfected cells did not produce any GM-CSF, and uP-mGM transfected cells produced 1,144±84 pg/ml. It is important to note that this concentration is from the supernatant after 72 h transfection, and transient expression after transfection usually has a large peak of protein expression in days between 2 to 5.

Usually, 2,000 pg/ml GM-CSF is used for macrophage polarization [1]. As the GM-CSF protein production is slow after transfection and probably reach this concentration in days 3 or 4 based on our result shown above, the lower number of iNOS positive cells in uP-mGM group is comprehensible.

Fluorescence intensity indicates that some cells expressing iNOS more than others, but comparing to the negative control groups, it is very clear the presence of fluorescence positive cells. Cell morphology shows that those cells with strong fluorescence are larger than non-fluorescent cells and similar to M1-like macrophages, and those cells with weak fluorescence are round-shaped and smaller, showing that they are under polarization process.

1. Weinstock LD, Forsmo JE, Wilkinson A, Ueda J, Wood LB. Experimental Control of Macrophage Pro-Inflammatory Dynamics Using Predictive Models. Front Bioeng Biotechnol. 2020;8:666. Epub 2020/08/09. doi: 10.3389/fbioe.2020.00666. PubMed PMID: 32766211; PubMed Central PMCID: PMCPMC7381235.


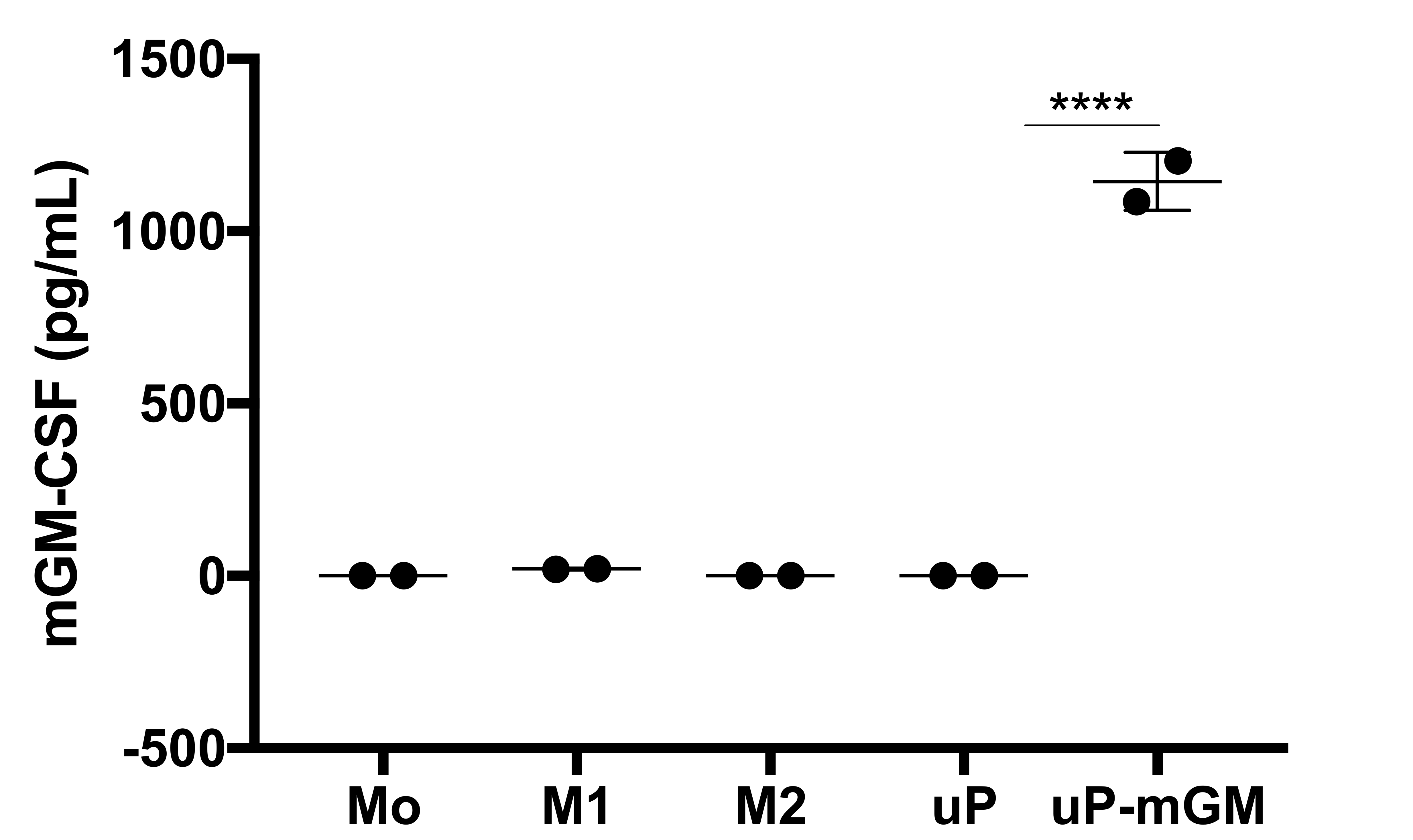


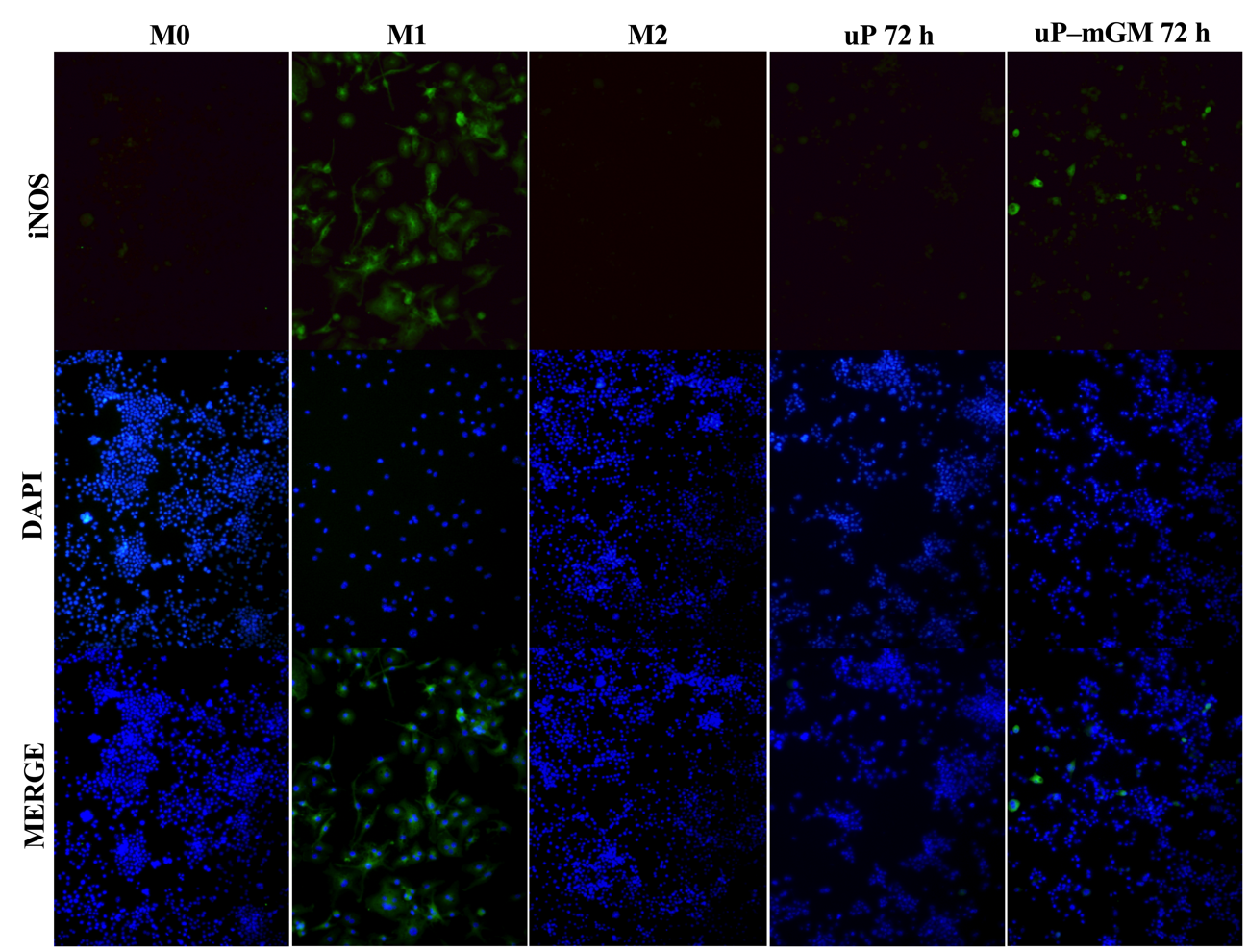

Supplement: Supplementary file 5 — Additional file 5. [file 13287_2020_1992_MOESM5_ESM.docx]
