# Supplementary material for: Skeletal muscle healing by M1-like macrophages produced by transient expression of exogenous GM-CSF
Source: Stem Cell Res Ther. 2020 Nov 6;11:473. doi: 10.1186/s13287-020-01992-1 (PMC7648431; doi:10.1186/s13287-020-01992-1)
Supplement: Supplementary file 6 — Additional file 6. [file 13287_2020_1992_MOESM6_ESM.docx]

**ADDITIONAL FILE 4**

**Calculation of GM-CSF concentration in the electroporated muscle**

In our previous study, we observed 8.6 ± 3.8 ng of GM-CSF in a thigh muscle weighing 160 ± 23 mg after electroporation with uP-mGM [1]. Considering that the muscle contains about 70% water, the water content in this muscle should be around 112 µL (160 mg x 70%). Therefore, GM-CSF concentration should be around 8.6 ng/112uL. The molecular weight of GM-CSF varies and depends on the degree of glycosylation, but assuming an average value as 30 KDa, the GM-CSF concentration should be around 2.6 µM (=0.08 g/L/30.000). As this concentration is well above 10 pM, in principle, the electroporation with uP-mGM should produce enough amount to stimulate hematopoietic cells proliferation, survival and functional activities.

However, there are several factors we should include in this calculation to approximate to the real concentration. For example, the value we found is an average value that should vary around the muscle, *ie*, at the transfected area this concentration should be higher than 10 pM which decreases gradually from this site. According to the Netter's Atlas of Human Physiology [2], about 1/3 of body water is from extracellular fluid, so the number we found above should be divided proportionally, at least. In addition, in the amino acid sequence of GM-CSF there is heparin binding domain [3], making it to be retained around the area where it is produced, which is a characteristic of paracrine factors. Probably, this is the main reason why we did not find GM-CSF in the circulation.

In summary, it is difficult to determine the real GM-CSF concentration in the transfected muscle, but as the value we estimated is much higher than 10 pM, it is reasonable to accept that there is enough amount of GM-CSF to stimulate biological activities by macrophages and other myeloid cells. A direct proof of this assumption is the increase of macrophages on day 4 after electroporation in the healthy (GM) and muscle injured mice (LM+GM) (Figure 5 b-c).

**REFERENCES**

1. Sacramento CB, Cantagalli VD, Grings M*, et al.* Granulocyte-macrophage colony-stimulating factor gene based therapy for acute limb ischemia in a mouse model. *J Gene Med* 2009; **11**: 345-353.

2. Hansen JT, Netter FH, Koeppen BM. Netter's Atlas of human physiology. ed). Icon Learning Systems: Teterbobo, N.J., 2002.

3. Sebollela A, Cagliari TC, Limaverde GS*, et al.* Heparin-binding sites in granulocyte-macrophage colony-stimulating factor. Localization and regulation by histidine ionization. *J Biol Chem* 2005; **280**: 31949-31956.
